# Post‐traumatic pleural effusion: Don't forget the chylothorax!

**DOI:** 10.1002/rcr2.1274

**Published:** 2024-01-05

**Authors:** Valentin Bovy, Tom De Keukeleire, Elke Van Schoote, Inge Vanslembrouck, Anne‐Marie Bogaert

**Affiliations:** ^1^ Department of Internal Medicine AZ Sint‐Elisabeth Zottegem Belgium; ^2^ Department of Pneumology AZ Sint‐Elisabeth Zottegem Zottegem Belgium; ^3^ Department of Internal Medicine and Geriatrics AZ Sint‐Elisabeth Zottegem Zottegem Belgium; ^4^ Department of Nephrology AZ Sint‐Elisabeth Zottegem Zottegem Belgium

**Keywords:** chylothorax, pleural effusion, thoracocentesis, trauma, triglycerides

## Abstract

In this case we describe a 58‐year‐old male with bilateral pleural effusion after a blunt trauma to the back. A pleural puncture revealed a chylothorax. An additional computed tomography scan showed a vertebral fracture at level D8 with rupture of the nearby thoracic duct. Our patient could be treated with a conservative approach. This case highlights the importance of ruling out a chylothorax in any post‐traumatic pleural effusion. Despite the low prevalence, we consider it a don't‐miss diagnosis given the specific treatment requirements.

## Graphical abstract

Most of the time, a post‐traumatic pleural effusion is linked by physicians to a hemothorax. A post‐traumatic chylothorax is usually not considered, largely because of the uncommon nature of this condition. In this article, we present a successful conservative treatment of a patient with post‐traumatic chylothorax after falling down the stairs.

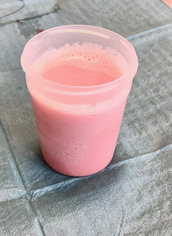

## INTRODUCTION

Most of the time, a post‐traumatic pleural effusion is linked by physicians to a hemothorax. A post‐traumatic chylothorax is usually not considered, largely because of the uncommon nature of this condition.

Because of the specific treatment requirements, we believe that a chylothorax should be suspected in any post‐traumatic pleural effusion. Diagnosis is made by pleural triglycerides level. Treatment is usually conservative, although surgical or percutaneous treatment may sometimes be indicated.

In this article, we present a successful conservative treatment of a patient with post‐traumatic chylothorax after falling down the stairs.

## CASE REPORT

A 58‐year‐old male presented to the emergency department after an accidental fall under the influence of alcohol. The patient fell backwards down the stairs causing trauma to his back. Both clinical and radiographic findings were reassuring, allowing the patient to leave the emergency department that same day.

**FIGURE 1:**
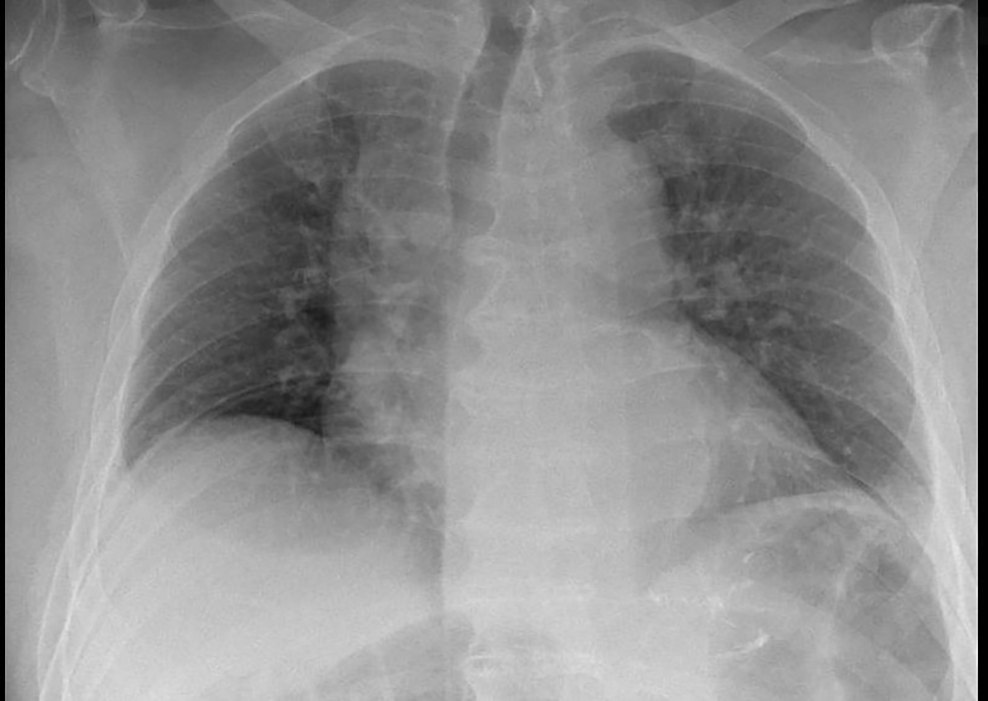
Chest x‐ray at the first emergency presentation.

**FIGURE 2:**
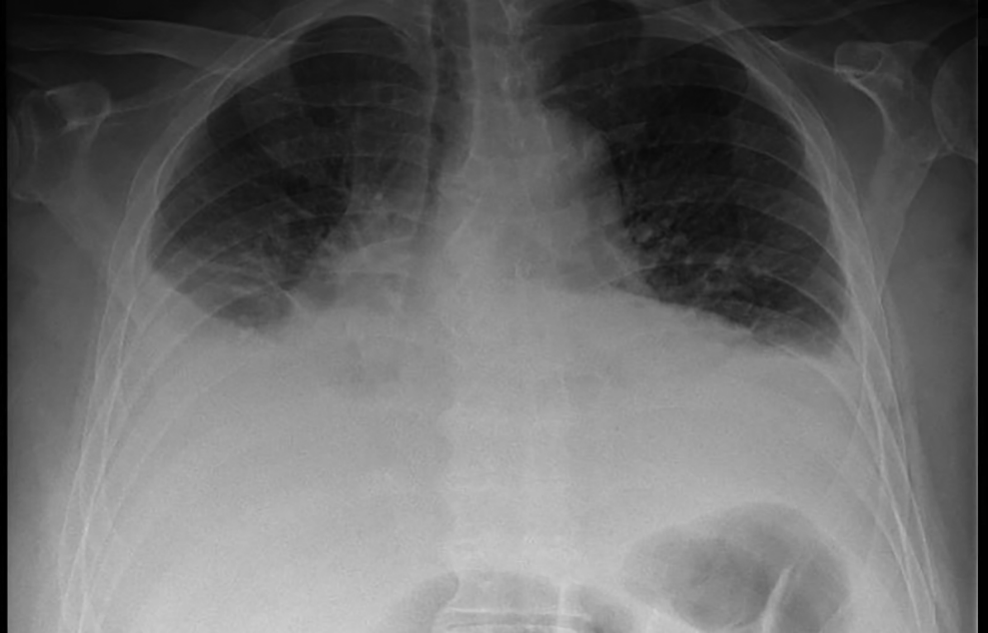
Massive pleural effusion 4 days later at the second emergency presentation.

**FIGURE 3:**
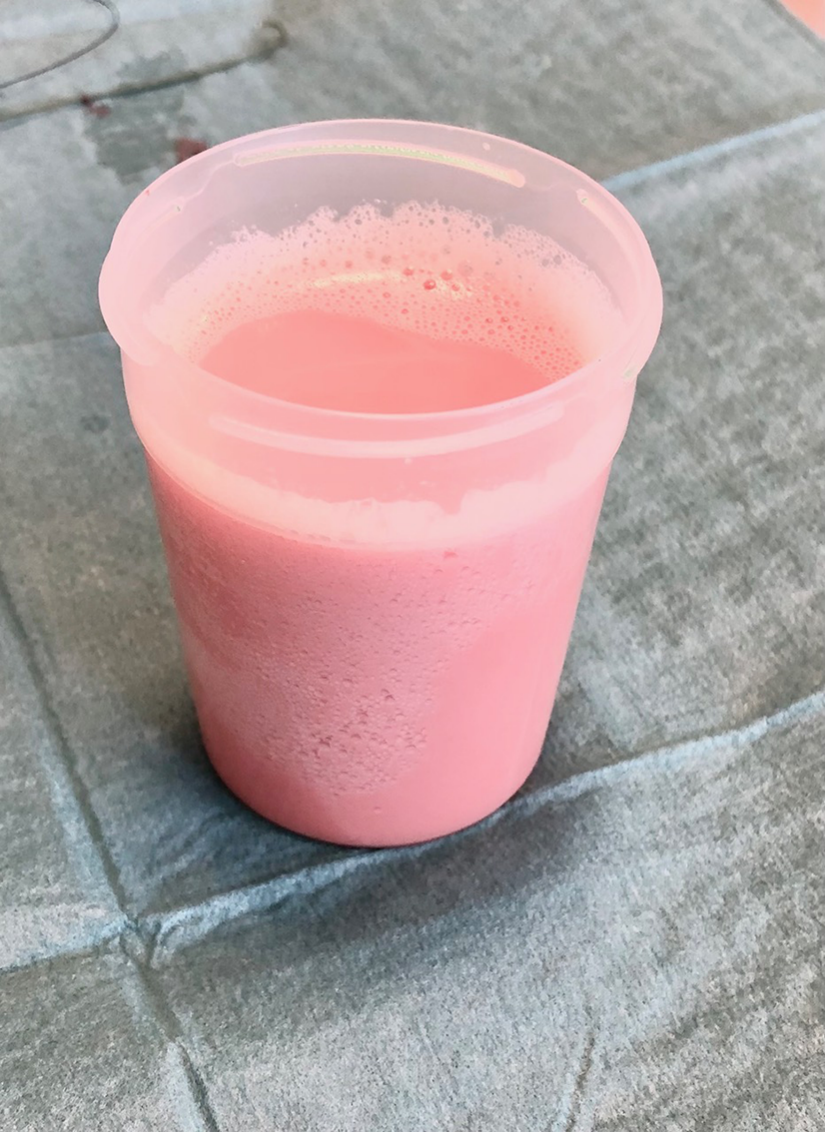
Through thoracocentesis a milky and slightly bloody fluid was evacuated.

**FIGURE 4:**
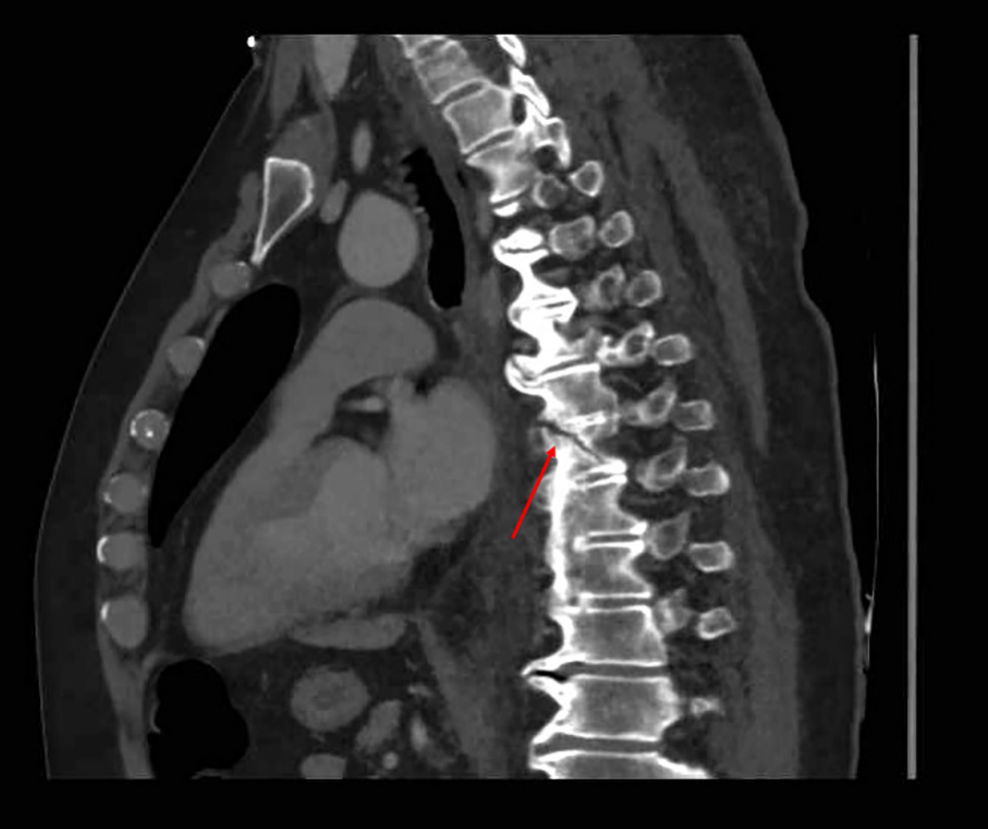
Chest computed tomography‐scan reveals an anterior vertebral fracture at level D8.

Four days later, the patient presented again at the emergency department. He reports an increased dyspnoea since his recent fall. Biochemistry shows mild inflammation (CRP 54 mg/L) without additional abnormalities. Clinically, we note a hemodynamically stable patient with markedly attenuated pulmonary auscultation in the lower lung fields. Chest x‐ray confirms a bilateral pleural effusion. Figures 1 and 2 show the chest x‐ray at the first and second emergency presentation, respectively. Since the fall occurred under apixaban, this pleural effusion was most suspicious for a hemothorax. We performed a diagnostic pleural puncture. A milky and slightly bloody fluid was evacuated (Figure 3). Analysis of pleural fluid showed an elevated triglyceride value of 3166 mg/dL (Table 1), indicative of chylothorax. An additional chest computed tomography (CT)‐scan was performed. The scan revealed a DISH pattern (Diffuse Idiopathic Skeletal Hyperostosis) with a recent fracture of the anterior vertebral body of level D8 (Figure 4).

**TABLE 1:** Pleural fluid analysis shows an exudative effusion with very high triglyceride level.

Pleural fluid analysis		
PH	7.34	
Protein (g/dL)	N/A	
Glucose (mg/dL)	127	
Amylase (U/L)	<30	[10–60]
LDH (U/L)	231	[75–148]
Cholesterol (mg/dL)	111	[150–190]
Triglycerides (mg/dL)	3166	[50–150]

The patient is known for Forestier disease, characterized by axial skeletal hyperostosis with typical ‘bamboo spine’. This abnormality makes the spine highly vulnerable to trauma. As a result of his fall down the stairs, the patient experienced hyperextension trauma of the back resulting in an anterior vertebral fracture. This vertebral fracture caused damage to the nearby thoracic duct resulting in a chyle leak with subsequent chylothorax.

We treated this patient by placing a bilateral chest drain. In addition, the patient was put on nil per os (NPO) to reduce the production of chylomicrons. Total parenteral nutrition (TPN) was started. The amount of evacuated chyle was closely monitored on a daily basis. Since it never exceeded the critical value of 1 L/24 h, we did not proceed to surgery. Under this treatment, we obtained a progressive reduction to complete cessation of chyle flow through the drains. Peroral feeding was therefore able to be restarted after 8 days: initially, a low‐fat and easily digestible diet after which we switched to a regular diet. As there was no recurrence of chyle flow, the drains could be safely removed. The total amount of evacuated chyle was 4.5 L on the right side and 0.8 L on the left side.

## DISCUSSION

A chylothorax results from damage to the thoracic duct (TD) or a collateral vessel of it. The TD runs from the cisterna chilli through the right side of the vertebral column to reach the left subclavian vein. However, there is considerable interindividual variation in the course of the TD. The TD is found to have multiple channels in 40% of cases, with significant variation.^1^ Even if a chylothorax will preferentially develop on the right side, this variation in anatomy explains why an effusion can also develop on the left side.

The main function of the TD is to drain lymphatic fluid back to the systemic circulation. In addition to its immunological function, lymphatic fluid is also involved in lipid transport. Fatty acid and cholesterol are packed within enterocytes as chylomicrons. These chylomicrons are too large for capillary absorption. Therefore, they are absorbed intestinally by the lacteales, which are the terminal branches of the intestinal lymphatic system. Following the above‐mentioned route, they finally reach the systemic circulation.^2^

To date, few large‐scale studies are available regarding the aetiology of chylothorax. A retrospective study of 203 patients shows that thoracic surgery (especially esophagectomy) is the most frequent cause of chylothorax (49.8%). Invasive malignancies (and lymphomas in particular) are in second place (16.7%). Exogenous blunt trauma as in our patient is described in only 1 of the 203 included patients, which corresponds to 0.4% of cases of chylothorax.^3^

The diagnosis of a chylothorax is made by analysis of pleural fluid. Typically, this is an exudative effusion with a milky appearance. Pleural triglycerides >110 mg/dL are generally accepted as a cutoff for the diagnosis of a chylothorax. Triglycerides <50 mg/dL exclude it. Retrospective review indicates a sensitivity of 86%.^4^ Three remarks need to be added here.

First, a retrospective study showed that only 44% of chylothorax had the typical milky appearance.^4^ The same study also found that 14% of patients presented with a transudative effusion. However, most of these patients don't have a traumatic aetiology as in our case.^5^

Second, it is important to note that examination of pleural fluid depends on the nutritional status of the patient. Fasting or malnourished patients will have low pleural triglycerides which may cause the diagnosis of chylothorax to be missed.

Last but not least, a pleural cholesterol level >200 mg/dL with (nearly) normal triglyceride level is suggestive for a pseudochylothorax (PCT). It is important to differentiate both since a PCT is more likely to occur in chronic inflammatory reactions and only rarely in trauma.^6^

In the last few years, nuclear medicine has also gained a place in the diagnosis of chylothorax. Lymphoscintigraphy using 99mTc—human serum albumin along with SPECT–CT is non‐invasive and showed promising results.^7^ This technique can be used for exact localization of the anatomical defect in the lymphatic vessels. For our patient, we decided not to proceed to nuclear investigation. Since the patient was treated in a conservative approach without surgery, there was no added value to identify the exact localization of the lesion.

Given the low prevalence of this disease, there is not yet a clear consensus on the management of chylothorax due to blunt trauma. Therefore, we are forced to rely on case reports. Recently published study of 39 cases showed that nearly three‐fourths of chylothorax due to blunt trauma could be treated with a conservative approach.^8^ Reducing peroral fat intake is essential to this. The rationale behind it is that the production of chylomicrons will decrease which will also decrease the leakage of lymph fluid into the thorax. In case of hemodynamic instability or if the flow of lymph fluid remains too high with a conservative approach, surgery is recommended. Again, there is no clear cut‐off regarding the maximum acceptable flow rate. We rely on recommendations coming from studies with postoperative chylothorax in which the limit of 1 L/24 h is adopted.^9^

Conventional surgical procedure consists of left‐sided posterolateral thoracotomy with ligation of the thoracic duct. However, less invasive procedures such as thoracic duct embolization (TDE) are on the rise. A study of 109 patients with traumatic chylothorax reported an overall success rate of 71% (77/109) with this technique.^10^ In addition, side effects were limited (3% of patients) and of a minor character. These findings are consistent with previous reports.^11^ Given high success rate and minimal complications, TDE can be considered as a valid alternative to invasive thoracic duct ligation.

## AUTHOR CONTRIBUTIONS

Each of the four authors had a significant and equivalent contribution to the conception and reviewing of this article. Each of them approves the published version.

## CONFLICT OF INTEREST STATEMENT

None declared.

## ETHICS STATEMENT

The authors declare that appropriate written informed consent was obtained for the publication of this manuscript and accompanying images.

## Data Availability

The data that support the findings of this study are available from the corresponding author upon reasonable request.
